# Solitary Plasmacytoma in the Mandible Resembling an Odontogenic Cyst/Tumor

**DOI:** 10.1155/2016/3629047

**Published:** 2016-12-19

**Authors:** Fatemeh Rezaei, Hesamedin Nazari, Babak Izadi

**Affiliations:** ^1^Department of Oral Medicine, School of Dentistry, Kermanshah University of Medical Sciences, Kermanshah, Iran; ^2^Department of Oral and Maxillofacial Surgery, School of Medicine, Kermanshah University of Medical Sciences, Kermanshah, Iran; ^3^Department of Pathology, School of Medicine, Kermanshah University of Medical Sciences, Kermanshah, Iran

## Abstract

A 46-year-old male patient referred to Department of Oral Medicine, with the primary chief complaint of a painless swelling in the right side of mandibular. A panoramic radiograph revealed a well-defined, multilocular radiolucent bony lesion with thin and straight septa in the right side of mandible extending from distal of canine to mesial of third molar. Histological examination showed a solid proliferation of atypical plasmacytoid cells, which was indicative of plasmacytoma. A systemic workup for the final diagnosis was performed to rule out multiple myeloma.

## 1. Introduction

The plasmacytoma is a neoplastic and monoclonal proliferation of plasma cells that usually arises within bones [1, 2]. Most of the lesions present centrally within a single bone, and it occurs most frequently in the spine, vertebrae, femur, and pelvis [3, 4]. Infrequently, it is seen in soft tissue, in which case, the term extramedullary plasmacytoma is used. The upper respiratory tract, especially the nasal cavity, oropharynx, nasopharynx, and sinuses, is frequently involved. It has a longer survival rate [5, 6]. However, the extramedullary plasmacytoma can convert to plasmacytoma of bone and myeloma, both of which are associated with a poorer prognosis [7, 8].

The male to female ratio of solitary plasmacytoma is approximately 2 : 1, with an average age of 55 years [9, 10]. The localization of solitary plasmacytoma of bone in head and neck is very rare and usually occurs in the sinonasal tract [11]. Approximately 12% to 15% of solitary plasmacytomas of the bone occur in the jaw and they are commonly involved in the posterior body of the mandible that can extend to angle and ramus [10].

## 2. Case Presentation

**Figure 1:**
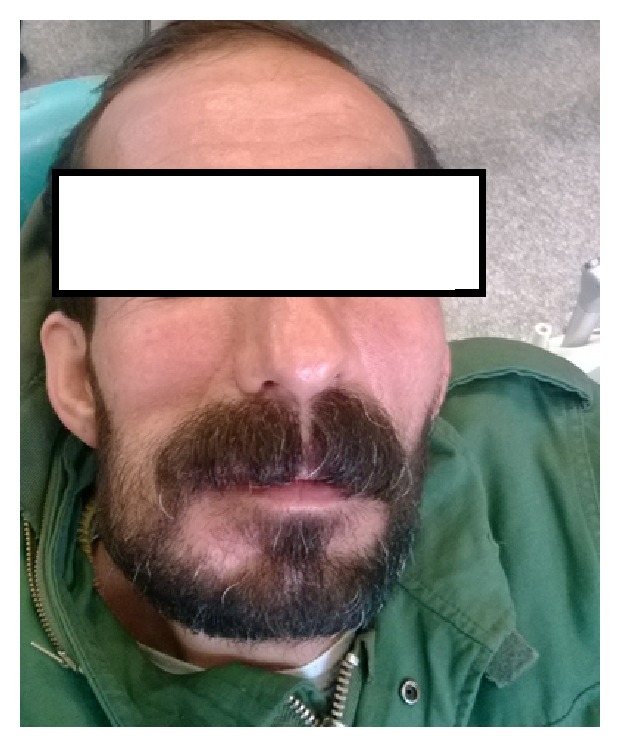
Swelling in the right side of mandibular bone.

**Figure 2:**
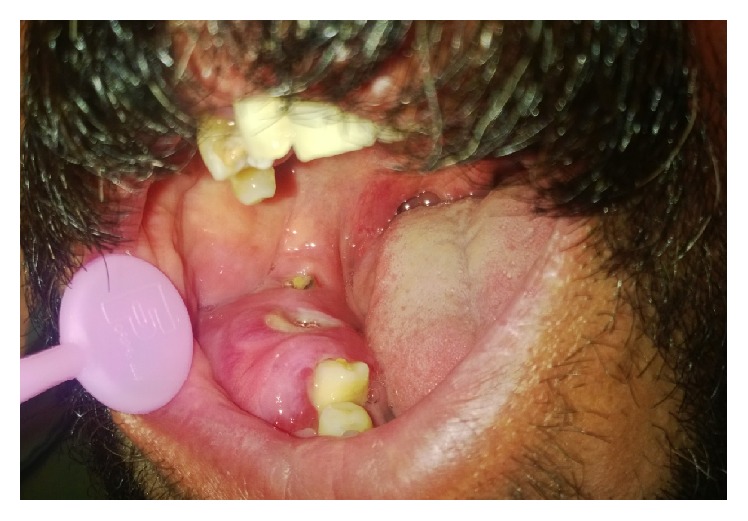
Clinical appearance of the expansion in the right side of mandible.

A 46-year-old male patient presented to the Department of Oral Medicine, Kermanshah University of Medical Sciences in 2015 with the primary chief complaint of a painless swelling in the right side of mandibular bone that he had first noticed 2 months before (Figure 1). He had medical history of epilepsy and seizures so he has consumed phenytoin and lamotrigine for about 13 years. He did not use tobacco, alcohol, or other intravenous drugs. His general health was good without fatigue, fever, or weight loss. Neurologic examination of cranial nerves V and VII was normal without visible skin changes or drainage. He had no complaint of paresthesia or anesthesia. Maximum opening of the mouth was 4 cm, without deviation or clicking on the temporomandibular joint. Intraorally, the involved area had firm consistency without tenderness and was covered by normal mucosa (Figure 2).

He had generalized periodontitis and bone loss. Second molars premolars were mobile (grade II) and oral hygiene was poor. The third molar of the right mandible was nonvital but the other teeth on the same side were vital. There was no evidence of palpable submandibular, submental, or cervical lymphadenopathy.

**Figure 3:**
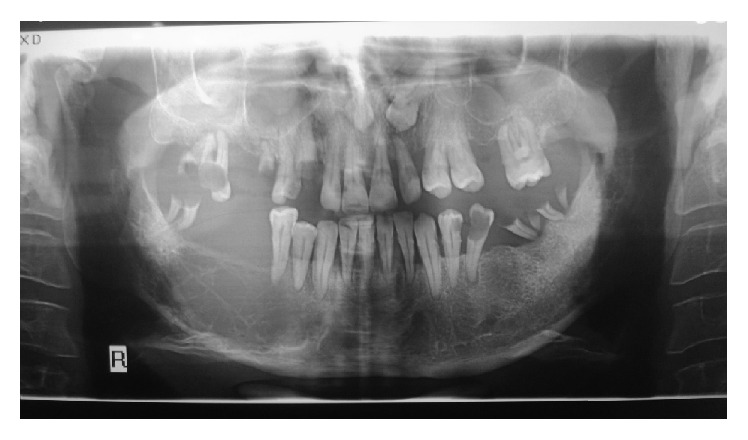
Panoramic radiograph demonstrating a large multilocular radiolucent lesion of the right mandible.

A panoramic radiograph revealed a well-defined, multilocular radiolucent bony lesion with thin and straight septa in the right side of mandible extending from distal of canine to mesial of third molar. Resorption of the roots of the adjacent mandibular teeth did not occur (Figure 3).

Magnetic resonance imaging (MRI) was ordered to reveal the invasion and destruction of the lesion to the soft tissues. It revealed an expansile destructive lesion measuring about 4.4*∗*6.3 mm is noted in right side of mandibular bone with extension to right side hypoglossus and mylohyoid muscles as well as outer subcutaneous fat and skin and right buccinators and master spaces.

### 2.1. Differential Diagnosis

An expansile multilocular radiolucent lesion in a middle-aged adult brings to mind a number of lesions that should be included in a differential diagnosis. The most common lesions based on the clinical manifestation and radiographic feature included odontogenic myxoma, ameloblastoma, and odontogenic keratocyst.

Odontogenic myxoma is an uncommon benign mesenchymal odontogenic tumor arising from the dental papilla, follicle, or the periodontal ligament [12]. It commonly involves the mandibular premolar and molar regions. It is most frequently seen in patients in the second to the third decades of life [13]. Odontogenic myxoma often grows without symptoms, most commonly presenting as a painless swelling [14]. Radiographically, its appearance ranges from unilocular to multilocular radiolucency with variable trabecular pattern giving rise to soap bubble, tennis racket, or honey comb appearance. Ideally, the septa that cause the multilocular feature are thin and straight, producing a tennis racket or stepladder pattern [15, 16]. Locating several straight septa in panoramic feature propelled us to this tumor as a first diagnosis.

Ameloblastoma is one of the most frequent odontogenic tumors [17]. It is most commonly seen in adults in the third to fourth decade but may be found in patients over a wide age range [18, 19]. Ameloblastoma most often presents as a hard painless intraoral swelling or as an incidental finding on routine dental imaging [20]. The radiographic features of conventional ameloblastoma are classified as unilocular or multilocular radiolucencies with well-defined borders [21].

The keratocystic odontogenic tumor (KOT) occurs in the 2nd and 3rd decade in the posterior body or in the ascending ramus of the mandible [22, 23]. The radiographic appearance of KOT may range from a small unilocular radiolucency to a large multilocular radiolucency. Multiple KOTs are associated with Nevoid basal cell carcinoma syndrome [24].

## 3. Diagnosis

Aspiration of lesion was nonproductive, so excluding the possibility of vascular and cystic lesions.

An incisional biopsy of the bony lesion was performed under local anesthesia without significant bleeding.

**Figure 4:**
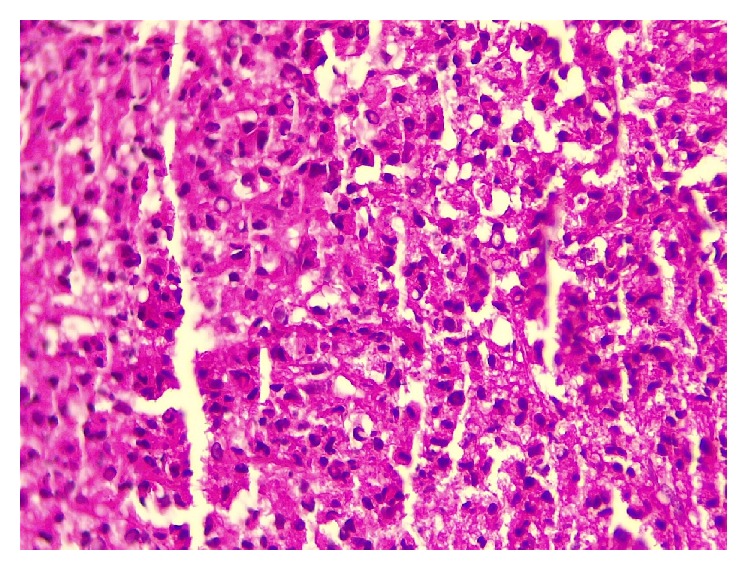
Diffuse infiltration of neoplastic large plasmablastic cells with pleomorphism and nuclei with one or several nucleoli (hematoxylin and eosin, original magnification ×40).

Histological examination on hematoxylin and eosin (H&E) staining showed a solid proliferation of atypical plasmacytoid cells with eccentric nuclei and basophilic cytoplasm, which was indicative of plasmacytoma (Figure 4).

In Complete Blood Count test (CBC and Diff test), white blood cell (WBC: 3600 cells/mL), red blood cell (RBC: 3.9*∗*10^6^ cells/mL), hemoglobin (Hb: 12.9 g/dL), and hematocrit (HCT: 36.6%) were low but Mean Corpuscular Volume (MCV: 92.9 fL) and platelet (171000/mL) were in the normal range. In biochemistry test, urea (37 mg/dL) and creatinine (1 mg/dL) were normal and hypercalcaemia was not found so renal insufficiency was ruled out. Serum immunoelectrophoresis showed an increase in M-protein [immunoglobulin (IgG) *κ* type] and also a decrease in albumin of plasma and albumin/globulin ratio was lower than normal.

A systemic workup for the final diagnosis was performed to rule out multiple myeloma. Radiographic survey including posteroanterior and lateral skull views was performed and showed no additional osteolytic lesion.

**Figure 5:**
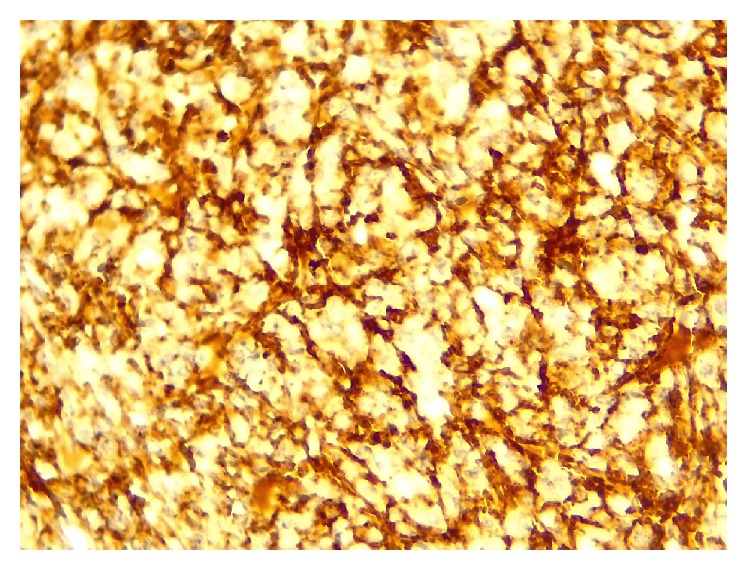
Immunohistochemical staining showing immunopositivity for CD138 (×40).

**Figure 6 fig6:**
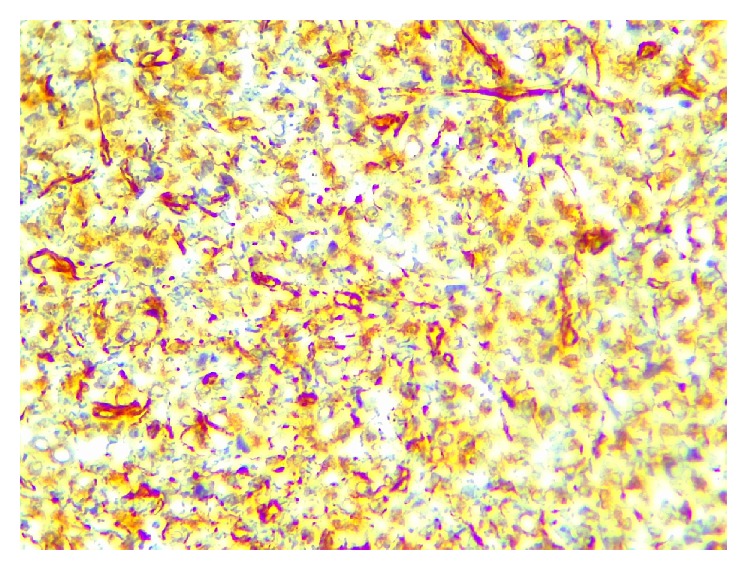
Immunohistochemical staining showing immunopositivity for vimentin (×40).

**Figure 7 fig7:**
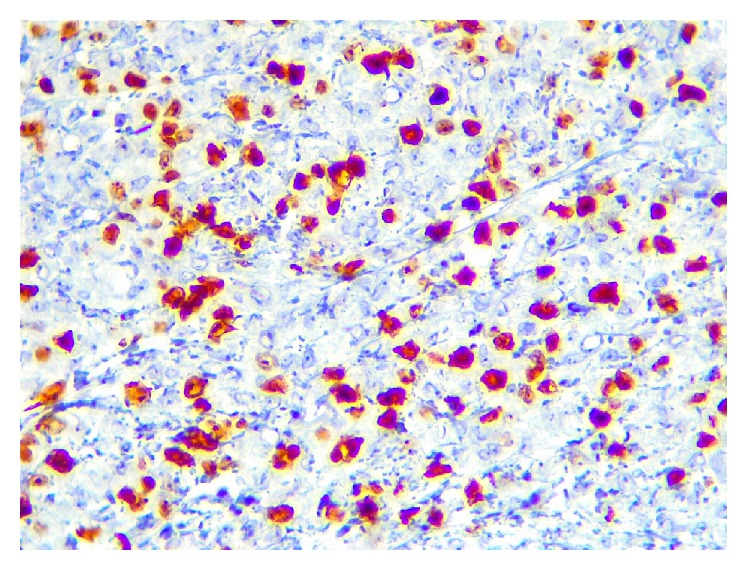
Immunohistochemical staining showing immunopositivity for Ki67 (×40).

**Figure 8:**
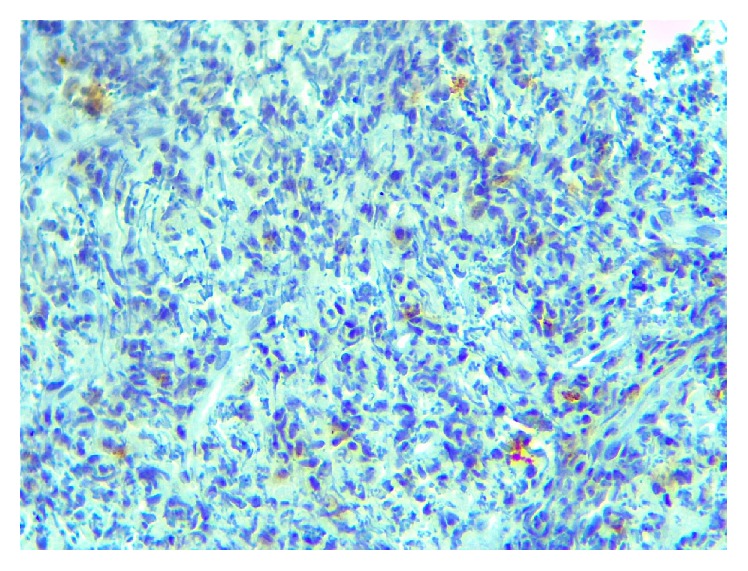
Immunohistochemical staining showing immunopositivity for EMA (×40).

In immunohistochemical painting, CD138, vimentin, Ki67, and EMA were positive (Figures 5 [Fig fig6] [Fig fig7]–8). But the immunohistochemical painting was negative for LCA, CK, CD3, CD20, CD1, NSE.

The patient was referred to haematooncologist. Bone marrow aspiration and trephine biopsy revealed hypercellular marrow with 6% plasma cell.

## 4. Discussion

Oral manifestations of solitary plasmacytoma of jaw include localized pain in the jaws and teeth, paresthesia, swelling, soft tissue masses, mobility and migration of teeth, hemorrhage, and pathological fracture. Fatigue and fever are the most common systemic symptoms [25, 26]. Our patient just had expansion in posterior mandible and mobility and migration of his teeth were probably due to aggressive periodontitis. He did not report history of pain or paresthesia in the jaws and teeth. Asymptomatic solitary bone plasmacytoma of the jaw is very rare but such a clinical form without pain has been described previously [27].

Solitary plasmacytoma of the mandible also has various radiographic findings: from well-defined, unilocular radiolucency or “punched-out” appearance similar to multiple myeloma (MM) to ill-defined destructive radiolucencies with ragged borders but without periosteal reaction [28–30]. The radiographic feature of the present case was well-defined, multilocular radiolucency with several straight septa that resembled odontogenic myxoma.

Diagnosis is based on the presence of malignant proliferation of plasma cells in the biopsy.

Histological features of solitary plasmacytoma are identical to MM, Sheets, or clusters of atypical monoclonal plasma cells with various types of differentiation [28, 29].

Bone marrow biopsies are performed to ensure the disease is localized. In solitary and extramedullary plasmacytoma, there will not be an increase of monoclonal plasma cells in bone marrow [28, 30]. Bone marrow aspiration in our patient revealed 6% plasma cell.

A typical antibody is composed of two immunoglobulin (Ig) heavy chains and two Ig light chains. A monoclonal protein (M-protein) is an abnormal immunoglobulin (IgG) light chain that is produced in excess by an abnormal clonal proliferation of plasma cells [31, 32]. Diagnosis of this present case was confirmed as solitary plasmacytoma, because laboratory examination showed the presence of M-protein [immunoglobulin (IgG) *κ* type] on serum electrophoresis. 25% to 50% of patients show a monoclonal gammopathy on evaluation by serum protein immunoelectrophoresis, although the amount of abnormal protein is much less than seen with MM reflecting the lower tumor burden [31, 32].

In immunohistochemical painting, CD138, vimentin, and EMA were positive. Also Ki67 was positive in 70% of tumoral cells. The results of the present case are in accordance with some studies who reported that these atypical cells react positively for CD138 and k-light chain, whereas staining with CD20, CD1, and NSE is essentially negative which ruled out epithelial, muscle, neural, histiocytic, and salivary gland origin of the tumor cells [6, 31].

An early diagnosis of solitary plasmacytoma of bone is essential to patient survival rate. It may represent the first manifestation of MM and conversion to MM occurring in about 70% of cases at an average of 20.7 months after initial diagnosis [28, 29]. So a systemic workup should be obtained early after diagnosis of the lesion to rule out the existence of this systemic disease [32, 33].

The patient did not show typical complications of MM, such as bone pain, skeletal destruction with osteolytic lesions, pathological fractures, limited mobility, hypercalcaemia, renal failure, and end-organ/tissue damage [34, 35].

CBC and Diff test showed WBC, RBC, Hb, and HCT were lower than normal range; however, this patient has consumed phenytoin and lamotrigine for about 13 years so these drugs can cause dyscrasias [36].

Solitary plasmacytomas are highly radiosensitive lesions. Radiation therapy, radical extensive surgery, or a combination of both is recommended as primary treatment. Radical radiotherapy comprising of 40–50 Gy has shown 80% of local disease control [9]. So the treatment used most commonly for both types of plasmacytoma is radiation therapy. Although chemotherapy is generally not used, treatment by local radiation and chemotherapy may delay converting it to multiple myeloma [37].

Surgery is rarely necessary but may be required in situations where plasmacytoma involvement of the bone causes skeletal instability and high risk of fracture. In these cases, radiation therapy may be delayed until after surgery. All patients with plasmacytomas require follow-up for at least the first five years after treatment has been completed. The course of solitary plasmacytoma of bone is relatively benign and the 5-year survival rate of it is 60%; however, it falls to 5.7% when progression to multiple lesions occurs [30].

The patient in the current case demonstrated partial response to radiation (40 Gy in 20 fractions) and chemotherapy (cyclophosphamide, hydroxydaunorubicin, and prednisone).

The disease remains stable and nonprogressive at this time. He is scheduled for at least 5-year follow-up appointments.

## 5. Conclusion

In this paper, we reported a male patient with a large destructive lesion in the posterior of mandible. It presented as an expansile multilocular radiolucent lesion, clinically and radiographically resembling an odontogenic cyst/tumor but it was solitary plasmacytoma.
